# Optimising the integration of technology-enabled solutions to enhance primary mental health care: a service mapping study

**DOI:** 10.1186/s12913-021-06069-0

**Published:** 2021-01-15

**Authors:** Haley M. LaMonica, Tracey A. Davenport, Antonia Ottavio, Shelley C. Rowe, Shane P. Cross, Frank Iorfino, Tanya A. Jackson, Michael A. Easton, Jennifer Melsness, Ian B. Hickie

**Affiliations:** 1 Brain and Mind Centre, The University of Sydney, 88 Mallett Street, Camperdown, NSW 2050 Australia; 2 InnoWell Pty Ltd, Shop 1-3/66-70 Parramatta Road, Camperdown, NSW 2050 Australia

**Keywords:** Mental health, Service mapping, Health information technology, Digital health solutions, Mental health services reform, Service performance indicators

## Abstract

**Background:**

Despite the widely acknowledged potential for health information technologies to improve the accessibility, quality and clinical safety of mental health care, implementation of such technologies in services is frequently unsuccessful due to varying consumer, health professional, and service-level factors. The objective of this co-design study was to use process mapping (i.e. service mapping) to illustrate the current consumer journey through primary mental health services, identify barriers to and facilitators of quality mental health care, and highlight potential points at which to integrate the technology-enabled solution to optimise the provision of care based on key service performance indicators.

**Methods:**

Interactive, discussion-based workshops of up to six hours were conducted with representative stakeholders from each participating service, including health professionals, service managers and administrators from Open Arms – Veterans & Families Counselling Service (Sydney), a counselling service for veterans and their families, and five *headspace* centres in the North Coast Primary Health Network, primary youth mental health services. Service maps were drafted and refined in real time during the workshops. Through both group discussion and the use of post-it notes, participants worked together to evaluate performance indicators (e.g. safety) at each point in the consumer journey (e.g. intake) to indicate points of impact for the technology-enabled solution, reviewing and evaluating differing opinions in order to reach consensus.

**Results:**

Participants (*n*=84 across participating services) created service maps illustrating the current consumer journey through the respective services and highlighting barriers to and facilitators of quality mental health care. By consensus, the technology-enabled solution as facilitated by the InnoWell Platform was noted to enable the early identification of risk, reduce or eliminate lengthy intake processes, enable routine outcome monitoring to revise treatment plans in relation to consumer response, and serve as a personal data record for consumers, driving person-centred, coordinated care.

**Conclusions:**

Service mapping was shown to be an effective methodology to understand the consumer’s journey through a service and served to highlight how the co-designed technology-enabled solution can optimise service pathways to improve the accessibility, quality and clinical safety of care relative to key service performance indicators, facilitating the delivery of the right care.

**Supplementary Information:**

The online version contains supplementary material available at 10.1186/s12913-021-06069-0.

## Background

### Mental health services reform

With the aim of achieving long-term, sustainable mental health reform, several governments have established strategic priorities specific to mental health care. In the United Kingdom, for example, NHS England and the Department of Health published Future in Mind, which highlighted the need for better access to crisis support, the integration of physical and mental health care, and early intervention and prevention [1]. These recommendations were echoed and elaborated on in a recent report from the Australian Government Productivity Commission that identified several target areas for reform, including: 1) prevention and early intervention for mental illness and suicide attempts; 2) expansion of services to close critical gaps in health care services; 3) provision of funding for services beyond health; 4) employment assistance for people with mental illness, including early intervention for work-related mental illness; and 5) commitment to fundamental reform to care coordination, governance and funding arrangements [2].

### Technology-enabled solutions for mental health care

Technology holds great promise for contributing to health care reform. For example, a review of randomised controlled trials of web-based interventions showed improvements in depression and anxiety symptoms as well as mental health literacy, reductions in the risk for eating disorders, and improvements in sleep for individuals with insomnia [3]. Furthermore, it has also been shown that ecological momentary assessment, or the repeated digital assessment of thoughts, feelings, and behaviours in real-time in association with a stated experience (e.g., therapeutic intervention), is more sensitive to change over time as it eliminates the need for retrospective reporting [4].

Despite the growing evidence base supporting the use of technologies for mental health care, the implementation of technology-enabled solutions in mental health services remains relatively rare. Certainly there are some notable exceptions as pointed out by Titov et al., who highlight the success of integrating Internet-delivered cognitive behavioural therapy (CBT) as part of clinical care with a mental health professional in five clinics internationally [5]. Our group has also had success in co-designing, implementing and evaluating technology-enabled solutions to support the mental health and wellbeing of young people, including for those attending university [6], living in disadvantaged communities [7], at risk of suicide [8], and seeking help at primary mental health services in Australia (i.e. *headspace*) [9]. Whilst these are examples of successful implementations of technology-enabled solutions, failures in the implementation of technologies as part of mental health care are prevalent. As summarised in our implementation science strategy [10], several studies have investigated barriers to successful implementation. Critical service-level factors include negative staff attitudes, staff resistance to change, and changes to work practices, such as increased workload [11, 12] as well as the importance of leadership from senior organisational and local service management to champion the technology [12, 13]. In a study of blended CBT care, or the provision of care through a combination of in-person and web-based sessions, the blended model of care was found to have equivalent treatment effects but poorer uptake by both consumers and health professionals and required longer treatment duration, thus resulting in higher costs [14]. Whilst the effectiveness of the web-based sessions may not have been equivalent to the in-person sessions, the researchers also suggested that the findings may relate to limitations in the implementation of blended care. Further research into the most effective ways to optimise routine mental health service delivery through the integration of technology-enabled solutions is still required.

### Service mapping

In recent years, health care systems have taken a lesson from the field of manufacturing to employ process mapping methods for the purposes of quality improvement [15]. Specifically, process improvement strategies have been defined as “systematic, organisation-wide programs that are adopted to improve quality and/or efficiency” [15]. Based on a review of eight quality improvement projects undertaken by the NHS, eight benefits of process mapping in health care were identified, including: 1) understanding of the complexity of the service or system; 2) identifying service gaps and opportunities for improvement; 3) engaging with key stakeholders; 4) aligning the project’s aims with the proposed intervention; 5) identifying leaders or champions to drive project progress; 6) learning collaboratively; 7) increasing empathy between stakeholders/professional groups; and 8) a tangible output – the process map [16]. Process mapping can be used in combination with other quality improvement techniques such as Lean, which emphasises waste reduction, improved customer outcomes, and greater return on investment [17]. However, as Lean has rarely been used to improve whole organisations, rather has typically been applied to discrete procedures or a single organisational process (e.g. accessibility of care), it has not been shown to be well suited to the complexities of mental health services due to the high variability in processes as well as difficulties in defining ‘waste’ [18, 19]. As such, we have chosen to apply process mapping as a distinct methodology in this study with the aim of better understanding the way in which to maximise the integration of technology-enabled solutions within mental health services in Australia to enhance the accessibility, quality and clinical safety of care. We have used the term ‘service mapping’ to refer to the structured approach to understanding the needs, existing processes, gaps in care, and performance of a mental health service as mapped against the consumer’s journey through the service. This information is then utilised to determine potential points of impact for the proposed technology in order to design a technology-enabled solution to optimise care.

### Objective

This study aimed to employ service mapping to: 1) illustrate the current consumer journey through a service from intake, assessment and treatment planning through to clinical review and service exit; 2) identify potential barriers to and facilitators of quality mental health care; and 3) highlight the potential points at which to integrate the technology-enabled solution as facilitated by a digital tool (i.e. the InnoWell Platform described in greater detail in the Methods section) within the service to optimise the provision of care based on key service performance indicators [20].

## Methods

### Design

This is a co-design study employing service mapping methodology (i.e. process mapping) to illustrate the current consumer journey through primary mental health services. As described by the World Health Organisation, primary mental health care is the first level of care within the broader mental health system, providing a range of services including the early identification of mental health symptoms and clinical trajectories, treatment of common mental health disorders, and referral to secondary and tertiary care as required [21].

### The InnoWell platform

In 2017, the Australian Government Department of Health and InnoWell (a joint venture between The University of Sydney and PwC [Australia]) entered into a three-year funding agreement to deliver Project Synergy (2017–20). As described by Hickie et al., Project Synergy’s objective is to develop and implement innovative health information technologies (including the InnoWell Platform) to enable improved mental health service delivery in Australia, facilitating better outcomes for people with lived experience, supportive others, health professionals and service providers [22]. As detailed in Davenport et al. [23] and Iorfino et al. [24], the InnoWell Platform is comprised of a multidimensional self-assessment targeting a range of biopsychosocial domains to capture a holistic view of the consumer. The assessment results are designed to be reviewed collaboratively by the consumer and their health professional to promote shared decision making in relation to care options, accounting for consumer preferences, as well as early identification of risk.

### Participating services and participants

Participating services included: 1) Open Arms – Veterans & Families Counselling Service (Sydney), a primary mental health counselling service for veterans and their families; and 2) *headspace* centres in the North Coast Primary Health Network (Coffs Harbour, Grafton, Lismore, Port Macquarie, Tweed Heads), primary youth mental health services. All staff involved in the implementation of the InnoWell Platform in participating services were invited to participate in the service mapping workshops including health professionals, service managers and administrators. Where staff from the services’ funding and/or governing bodies (i.e. service providers and/or lead agencies) were associated with implementation, these staff were also invited to participate. This wide-ranging recruitment is critical to ensure that data are captured from stakeholders at all levels of participating services. The Participant Information Sheet describing the study in more detail was distributed via email to eligible participants by an implementation officer embedded within the services. At the beginning of each workshop, the facilitators provided the participants with an opportunity to ask questions and clarify details of the research before providing written informed consent. Importantly, each participant was explicitly told that their participation was voluntary and they were free to withdraw from the workshops at any time without consequences. There were no formal inducements for participating in the workshops; all sessions were conducted during business hours, thus participants were paid as part of their normal work day.

### Service mapping

Service mapping workshops of up to 6 h in duration, including breaks and lunch, were conducted with each participating service. The workshops were coordinated by two facilitators, at least one of whom was a mental health professional. A scribe was present to take notes throughout each workshop. As previously described in LaMonica et al. [10], the interactive, discussion-based service mapping workshops were conducted with representative stakeholders from each participating service, including health professionals, service managers and administrators, in order to co-design the optimised technology-enabled solution for service delivery. In the first half of the workshop, the pre-existing service pathway (i.e. consumer’s journey through the service) and related staff roles and responsibilities were mapped, including intake, initial assessment, treatment planning and intervention, routine outcome monitoring, and service exit (or discharge). Gaps in current service delivery were reviewed relative to key features of high-quality mental health care to identify areas for improvement or reform as identified by the respective services (i.e. improved suicide risk identification, reduced wait times at intake) [10]. Service quality is an overarching concept that includes eight interrelated and internationally adopted domains shown in Table 1 [20]. Each of the eight safety and clinical quality domains were mapped to the point of interest on the consumer journey that may be enhanced via the technology-enabled solution. In other words, the workshop participants might first identify barriers to service access at the point of intake and then hypothesise how the technology-enabled solution could be implemented to improve this aspect of care.

**Table 1:** Key performance indicators of high-quality mental health care

Performance indicator	Example metric for measurement
Clinical safety	How suicide risk is assessed and mitigated at service entry
Accessibility	Ease of access for high risk sub-populations
Effectiveness	Proportion of users who return to work or education
Acceptability and satisfaction	Consumer satisfaction with care
Efficiency	Cost-effectiveness of care
Appropriateness	Matching service provision to clinical stage, which is an adjunct to mental health diagnosis that incorporates illness severity and risk of progression to facilitate appropriate treatment matching
Continuity and coordination	Successful transitions from primary to secondary care
Workforce competence and capability	Assignment of skilled staff to specific interventions

In the second half of the workshop, a technology-enabled solution was then co-designed, intertwining established service processes with the additional technology elements and processes (e.g. web-based initial assessment, clinical and non-clinical care options) with an emphasis on enhanced service provision as measured by identified service performance indicators. Importantly, participants from multiple services were included in this study to highlight the utility of service mapping as a means by which to illustrate the variability in the consumer journey through individual primary health services. Service mapping allowed for the identification of unique elements of the consumer flow (i.e. by whom is the referral and intake managed, how are consumers allocated to appropriate health professionals, etc.) as well as barriers to and facilitators of quality care for the respective services. A sample service mapping workshop agenda is provided in Additional file 1.

### Data collection and process map development

To align with how process mapping methods have been applied previously in the health service literature, the facilitators worked collaboratively with participants to map the current consumer journey through the respective participating services and then overlay the intersection and impact of the InnoWell Platform at key points in care (e.g. referral, assessment, treatment planning, intervention, review, and service exit) [25]. The service map was also reviewed from the perspective of health professionals working with consumers, including review of the data collected via the InnoWell Platform as well as in-person sessions, and service managers to understand the impact of the clinical digital tool at all levels of service delivery. Maps were drafted on a white board or butchers paper sheets, allowing for real time review and refinement during the workshops. Following the workshop, one of the facilitators created a final electronic version of the service map. Participants also worked together to identify the potential technology-enabled impacts on key performance indicators at each step of the process. Through both group discussion and the use of post-it notes, participants evaluated each service performance indicator (e.g. safety) relative to each point in the service pathway (e.g. intake). Differing opinions were reviewed, discussed, and evaluated by the participants, ultimately resulting in agreement by consensus. Importantly, decisions were reached by the participants and were not directed by the facilitators.

## Results

### Participants

Twenty-one staff members from Open Arms – Veterans & Families Counselling (Sydney), including 20 health professionals and 1 service manager, and 63 staff members from five *headspace* centres, including 43 health professionals, 7 service managers and 13 administrators, consented to participate in the service mapping workshops at their respective services.

### Service maps

**Fig. 1:**
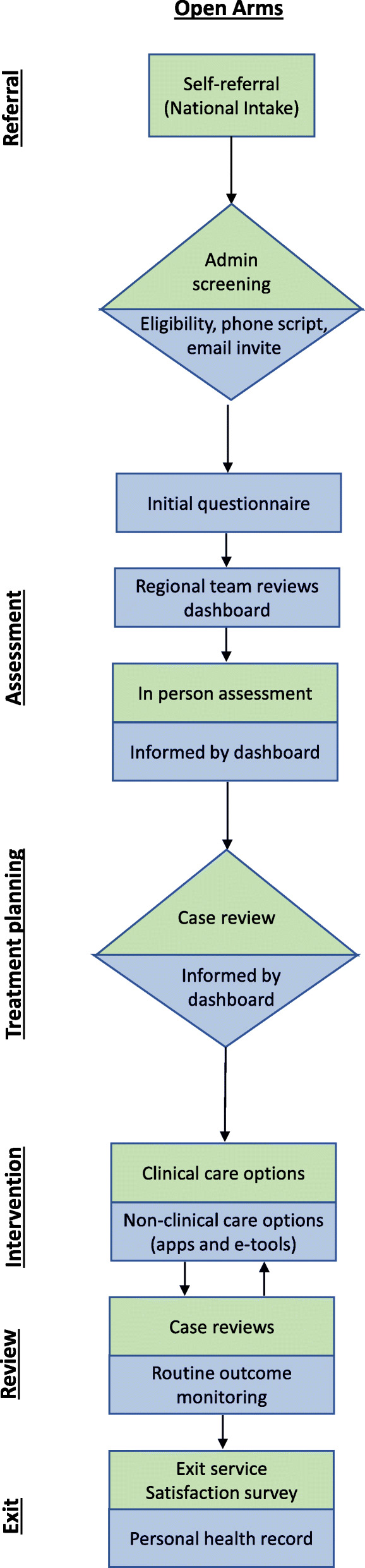
Technology-enabled service map for Open Arms

**Fig. 2:**
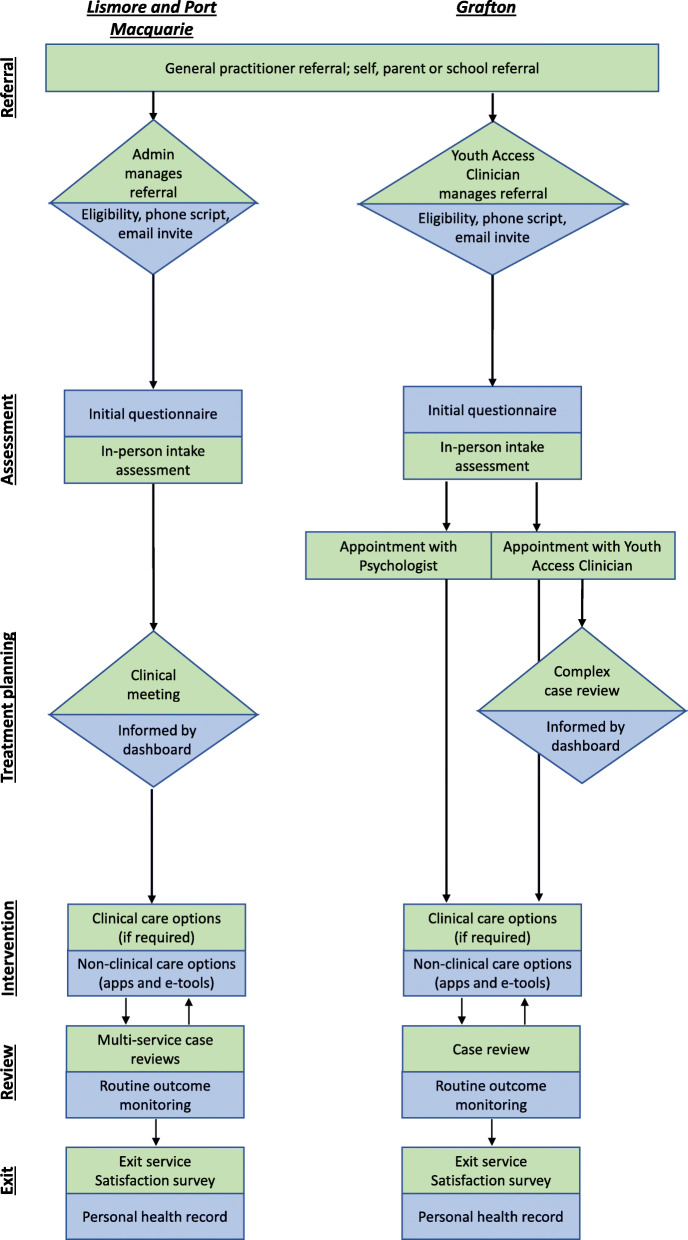
Technology-enabled service map for *headspace* Lismore, Port Macquarie and Grafton

**Fig. 3:**
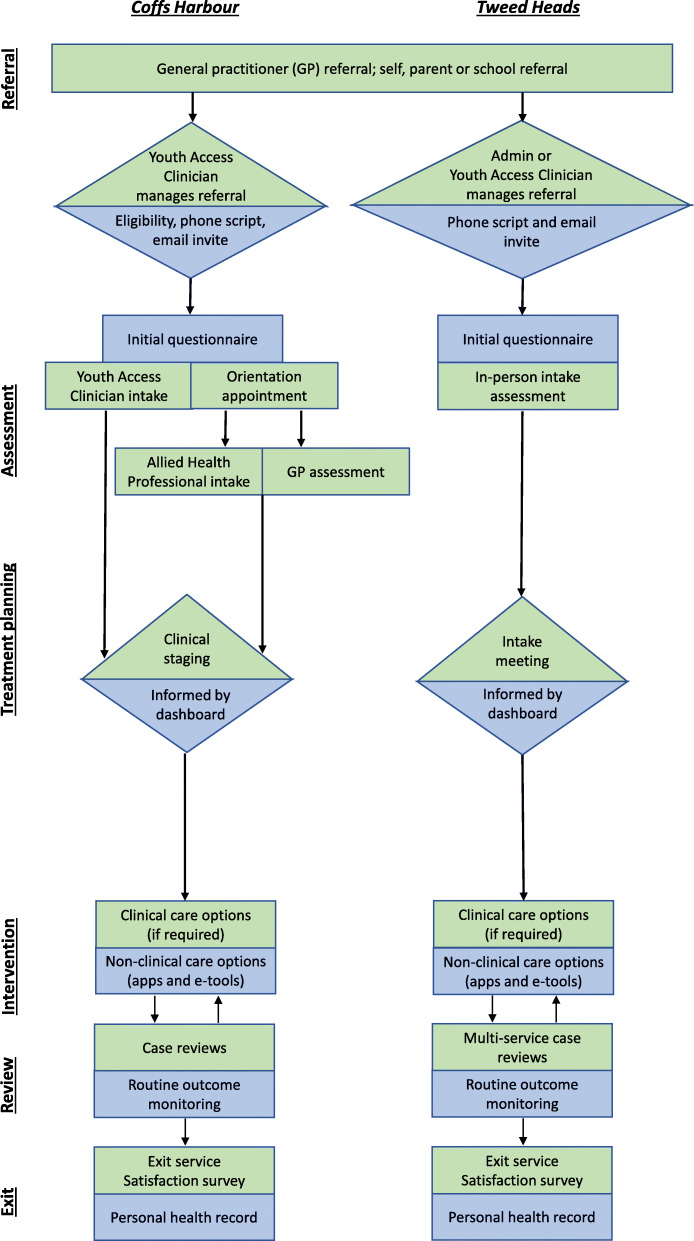
Technology-enabled service map for *headspace* Coffs Harbour and Tweed Heads

Standard process mapping symbols have been used in the service maps presented below, including rectangles to indicate a task that needs to be completed, arrows to represent the consumer’s journey in the service, and diamonds to represent decision points (see Figs. 1, 2 and 3). Additionally, green reflects the existing service pathway, whereas blue indicates the impact of the technology.

The initial questionnaire refers to the multidimensional, self-report, web-based assessment facilitated by the InnoWell Platform [23]. To ensure appropriateness for the consumer-base, each respective service implementing the InnoWell Platform has the option to select from a library of evidence-based psychometric tools frequently used in clinical practice (e.g. Kessler Psychological Distress Scale, Patient Health Questionnaire-9, etc.) [24]. Depending on the services selections, the initial questionnaire is expected to take up to 45 min to complete.

### Defining standard service delivery

Service mapping provides a critical opportunity to understand how the technology-enabled solution facilitated by the InnoWell Platform could be embedded within standard service delivery for each of the participating services. More specifically, participating services have the opportunity to implement the InnoWell Platform in varying ways within their service delivery models. At Open Arms, for example, it was determined initially that consumers would be invited to the InnoWell Platform by their health professional, either when scheduling their first appointment or thereafter based on clinical judgment. Due to the burden on the health professionals, the invite was later transferred to the administrative staff and included as part of the initial eligibility screening process, with consumers being asked to complete the multidimensional assessment (i.e. the initial questionnaire) prior to attending the first session with a health professional.

Whilst some variability in the invite process was evident across the *headspace* centres, reflecting the diversity in service models, young people were invited to the InnoWell Platform either by an administrative staff member or by a youth access clinician at the initial point of contact with the service or as part of community outreach programs. The young person was encouraged to complete the multidimensional assessment prior to completing the *headspace* specific intake assessment at which time they would be allocated to a health professional based on clinical stage (for those services employing staged care) and/or level of need. Across all services, the InnoWell Platform can then be used collaboratively by the consumer and their health professional to track and monitor outcomes over time and, in turn, modify treatment plans as needed if the initial approach proves ineffective (i.e. outcomes remain unchanged), the clinical presentation worsens, or the consumer’s circumstances change.

### Potential impact on key performance indicators

At a high-level and as agreed upon by consensus, the service mapping workshops served to highlight potential points of impact of the technology-enabled solution based on key performance indicators. In relation to *clinical safety*, participants noted that the multidimensional assessment had the potential to improve the early identification of suicide risk, thus enabling fast tracking into care. Whilst all services had a procedure in place to identify risk of suicide (e.g. consumers indicating distress when contacting Open Arms are immediately connected to a health professional), including referrals to appropriate specialist services thereafter, participants recognised that a digital tool had the potential to respond in real time rather than as determined by service operating hours and health professional availability.

Participants also recognised the considerable clinical value of the InnoWell Platform’s multidimensional assessment. Participants from Open Arms, for example, noted that the assessment would replace the need for the required telephone intake which, at the time of this workshop frequently was associated with a wait time (the service model has subsequently been redesigned so that intake assessments are offered at the time of the initial contact with the service [26]), thus improving *accessibility.* Similarly, participants from *headspace* indicated that the assessment could identify the need for and support the drafting of a mental health plan to enable timely access to specialist care. It was also recognised that a uniform and service-specific initial assessment had the potential to streamline the assessment process, reducing or eliminating the variable and, at times, lengthy intake process, resulting in improved service *efficiency.*

The use of reliable psychometrics validated for the target consumer base was also thought to improve *effectiveness* by facilitating the assessment of a consumer’s clinical presentation and daily functioning rather than relying solely on clinical opinion. Furthermore, routine outcome monitoring of a consumer’s treatment response over time as evidenced by changes in symptom severity, functioning and wellbeing assessed via the self-report assessment in the InnoWell Platform, would serve to rapidly identify when a treatment approach was not effective, thus facilitating change, including potential change in care team or referral to more intensive specialist care.

Whilst not an electronic medical record, it was recognised that the InnoWell Platform provides a comprehensive treatment record both for consumers (i.e. personal health record) and health professionals to inform referrals to specialist services as well as future episodes of care, thus enabling *continuity of care* and *care coordination*. In other words, the consumer retains a full record of their data from the InnoWell Platform which can then be shared with other health professionals, preventing the need to retell their story and start again from the beginning should changes occur or be required in their care team.

With regard to *acceptability,* it was noted that the InnoWell Platform serves as a clinical toolbox for both health professionals and consumers, ensuring both are aware of and informed about available care options. In this way, the InnoWell Platform positions the consumer at the centre of care. Additionally, participants from both Open Arms and *headspace* concluded that consumer *satisfaction* could easily be assessed via a simple and user-friendly survey delivered via the InnoWell Platform, with automatic prompts to facilitate completion.

Finally, it was highlighted by participants from Open Arms that the InnoWell Platform would facilitate communication between health professionals, facilitating joint case reviews and supervision, thus improving *workforce capacity*. The library of clinical resources available as part of the InnoWell Platform, including clinical training guides, factsheets, links to reputable organisations and websites, and apps and etools, also were noted to have the potential to support professional development.

## Discussion

### Technology-enabled enhancements in care based on key performance indicators

The service mapping process served to engage key stakeholders at the respective services to identify gaps in care and opportunities for improvement at each step in the consumer’s journey as well as to align the aims of implementing the technology-enabled solution with the services’ goals for reform. Improved clinical safety reflects one of the primary aims of the implementation of the co-designed technology-enabled solution within both Open Arms [26] and *headspace* services [27]. Importantly, the InnoWell Platform’s suicide escalation protocol is designed to facilitate risk identification and detection of suicidal thoughts and behaviours [23, 28]. Although the potential for new technologies to aid in suicide prevention is widely recognised, such tools are frequently under-utilised [29] and have not been rigorously evaluated for effectiveness [30]. Nevertheless, studies have shown that consumers are more likely to disclose suicidal ideation via web-based approaches rather than in-person assessment [31, 32], thus facilitating intervention, which underscores the importance of optimising implementation within standard service delivery.

In addition to facilitating disclosure, web-based assessments have good concordance with traditional paper-and-pencil measures [33] and clinician-based structured interviews [34] and are associated with lower costs and greater convenience for consumers. Importantly, our team’s previous work shows that web-based assessments are both more efficient than in-person clinical assessments and enhance the subsequent intervention sessions, enabling health professionals to focus on the delivery of interventions matched to a consumer’s level of need [35]. Additionally, the systematisation of the assessment and monitoring process ensures transparency of outcomes for both consumers and health professionals, which is particularly relevant as health professionals tend to overestimate the benefits of care relative to measured outcomes [36]. Importantly, our own research has shown that the ability to track data over time is valued by consumers as a means to better understand what information, resources and intervention strategies are associated with positive health outcomes [37]. Furthermore, symptom tracking and real-time feedback improves engagement and promotes behaviour change [38].

Whilst consumer satisfaction with service delivery, including in this case technology-enabled care, has been shown to be associated with clinical factors, such the presence of major mental illness (i.e. psychosis, bipolar or unipolar depression), it is also impacted by age and general quality of life (i.e. housing, social connections, financial circumstances, etc.) [39]. Nevertheless, a systematic review highlighted that consumer experience (i.e. satisfaction) is positively correlated with both self-rated and objectively measured health outcomes as well as treatment adherence and engagement with preventative care options [40] and as such, consumer satisfaction is increasingly valued as an indicator of service performance. With that being said, there is neither a consensus on how to define satisfaction nor a gold standard assessment measure.

### Limitations

This study has some limitations that are important to note. As participants were recruited from all levels of participating services, there is the possibility that some participants may have perceived there to be an unequal relationship. Importantly, participation was voluntary; therefore, no staff members were required to attend or contribute to the workshops if they felt uncomfortable. Furthermore, the participation of staff members with differing roles within the services was not raised as an issue at any time (the contact details of the researchers was available on the Participant Information Sheet). The understanding of how the technology-enabled solution might impact workforce capacity requires further exploration and reflects a limitation of this study. It is our hypothesis that the use of data analytics embedded within technologies will allow health professionals to examine their clinical performance based on consumer outcomes and in relation to consumer demographics, mental health disorders, stage of illness, and intervention method, thus identifying areas of strength as well as areas that would benefit from training, professional development, or supervision [41]. Additionally, it is important to note that the co-design process was undertaken without contributions from consumers with lived-experience of mental ill health. Our group recognises the importance of including the voice of those with lived experience in mental health service reform efforts, and aim to include consumers in all aspects of our research related to the design, development, implementation and evaluation of health information technologies.

## Conclusions

Service mapping, a processing mapping methodology, was shown to be an effective tool to engage key stakeholders to understand existing service pathways and, in turn, to identify barriers to and facilitators of quality mental health care. In this study, the resultant service maps highlighted potential points at which the technology-enabled solution could be embedded in the process to drive improvement on key performance indicators. The use of service mapping prevents a one-size-fits-all approach to service reform, but rather takes into account specific factors impacting the quality of care at each individual service, thus facilitating the delivery of the right care first time.

### Future directions

Whilst service mapping identified potential ways in which to enhance care based on key performance indicators at a high-level, it is now essential that the hypothesised technology-enabled service models be implemented and rigorously evaluated. To that end, the co-designed technology-enabled solutions as facilitated by the InnoWell Platform have been implemented in four of the above mentioned *headspace* services (Coffs Harbour, Lismore, Port Macquarie, and Tweed Heads) as well as Open Arms (Sydney and Lismore). As described in LaMonica et al. [42], we are using web-based surveys, semi-structured interviews, workshops, observational logs, and service audit data to gain valuable insights as to the impact of the implementations on consumer outcomes, health professional practices, and key service performance indicators as well as social return on investment (i.e. social, environmental, and/or economic value) [43].

Additionally, our team has further developed our service mapping methodologies by applying systems science approaches that makes use of dynamic simulation modelling. This recognises that services represent a collection of individual agents (e.g. patients, clinicians) whose actions are dynamic and interconnected, which often gives rise to common features of complex systems, such as nonlinear relationships, feedback loops and contextual effects [44, 45]. Dynamic simulation modelling is a technique which allows us to create an in-silico laboratory whereby we can model existing service pathways (derived from service mapping), the roles of various agents within these pathways and the role of technology to test proposed changes and their potential impact in a safe (simulated) environment before implementation. Simulations can be run multiple times using different input parameters or assumptions so that service administrators can compare outcomes under a range of hypothetical scenarios. This provides a systems perspective of the service and facilitates a deeper understanding of which factors may influence the success or failure of any given implementation. Agent-based modelling is one such approach which allows us to model system behaviour in terms of the agent-to-agent interactions (e.g. patient to clinician, or service to patient), and discrete event modelling allows us to transform the existing and proposed service maps into resource-constrained processes so that we can more accurately understand how staff resourcing and service pathways influence service efficiencies and patient outcomes within the complex mental health system [46]. The resulting simulation model can be used as a dynamic tool to guide decision-making about implementation, to set implementation targets and track progress against an ‘ideal’ benchmark by supplementing it with new data during and post implementation.

## Supplementary Information

**Additional file 1.** This file documents a sample agenda for conducting a service mapping workshop as outlined in the Methods of this paper.

## Data Availability

The data used and analysed during the current study are available from the corresponding author on reasonable request.

## Abbreviations

BMC: Brain Mind Centre
DOH: Department of Health
HREC: Human Research Ethics Committee
PHN: Primary health network
OPC: Outreach program counsellors
