# Supplementary material for: Optimising the integration of technology-enabled solutions to enhance primary mental health care: a service mapping study
Source: BMC Health Serv Res. 2021 Jan 15;21:68. doi: 10.1186/s12913-021-06069-0 (PMC7811218; doi:10.1186/s12913-021-06069-0)
Supplement: Supplementary file 1 — Additional file 1. This file documents a sample agenda for conducting a service mapping workshop as outlined in the Methods of this paper. [file 12913_2021_6069_MOESM1_ESM.docx]

**Service Mapping Workshop**

**Agenda**

**Date:**

**Time:**

**Location:**

**University of Sydney Facilitator**:

**University of Sydney Scribe:**

**Counselling Support:**

**InnoWell Attendees:**

**Materials required**

- Participant Information Sheets
- Participant Consent Forms
- Pens or pencils
- Sharpies
- White board markers
- Post-it notes
- A3 paper
- Tape

**AGENDA ITEMS**

**1:00pm Introduction**

- Informed consent
- Introductions – Name and role
- Overview of workshop
  - Understand the current service model
  - How does the technology integrate and impact upon the model?

**1:10pm Now: Service Map**

- **Provide a sample service pathway model to the group as an illustration of what we are trying to accomplish**
- Current service model – Using a white board, draw the service model across each service type (e.g. outpatient, inpatient, aged care, home visits)
  - How are referrals received in your service?
  - Who manages the referrals?
  - What happens when a referral is received from a service provider? Is this different when a partner or carer calls?
  - What is the process to obtain an appointment?
  - What does the intake and assessment involve?
  - Who completes the intake?
    - What if the individual is unable to complete the intake? Will this be done with a carer or informant?
  - What is the average time from referral to intake and then from intake to an appointment?
  - How is it determined which service an individual will enter and which health professional(s) they need to see?
  - Do individuals transfer between services? How does this occur?
  - Given this is such a multidisciplinary service, is there a standard process for treatment planning? Length of service?
  - What interventions are provided by your service – does this differ across sites?
    - Are there different wait times for each intervention?
  - Does your service have a mechanism for case review?
    - If yes, how does this occur, who is involved?
  - How are clients discharged from the service?

**If we have time:**

**Future: Service Mapping - Exploration of how the technology-enabled solution could enhance the service**

- **Provide a sample technology-enabled service model with impacts to the group as an illustration of what we are trying to accomplish**
- Confirm service model as discussed above
- Where does the technology impact on this pathway?
  - Who will offer the Platform?
  - When will it be offered to users?
  - Will both new and existing patients be offered the Platform?
  - Who do you anticipate completing the questionnaires (e.g. individual, carer, or with assistance)?
    - If an individual cannot answer the questions independently or with assistance, how would the information be captured (e.g. same questionnaires completed by a carer? Questionnaires explicitly addressed to the carer about the individual?)
      - *If the latter, we will need to consider informant questionnaires to create a proxy questionnaire.*
    - Who will assist the client across service settings?
  - How will the information be used in the service? Does this differ by service setting?
  - What is the expectation regarding ongoing use of the Platform by individuals as part of their care for tracking purposes?
  - What follow-up will occur if a patient has not accepted the invite or completed the questionnaire?
  - Who will discharge clients from the Platform? How will this decision be made?
- What data will need to be transferred to CHIME? If there is not an automated solution, how will this occur?

**Confirm next meeting**
